# Reviewing explore/exploit decision-making as a transdiagnostic target for psychosis, depression, and anxiety

**DOI:** 10.3758/s13415-024-01186-9

**Published:** 2024-04-23

**Authors:** Alex Lloyd, Jonathan P. Roiser, Sarah Skeen, Ze Freeman, Aygun Badalova, Adeola Agunbiade, Chuma Busakhwe, Carla DeFlorio, Anna Marcu, Heather Pirie, Romana Saleh, Theresa Snyder, Pasco Fearon, Essi Viding

**Affiliations:** 1 Clinical, Educational and Health Psychology, Psychology and Language Sciences, University College London, 26 Bedford Way, London, WC1H 0AP UK; 2 Institute of Cognitive Neuroscience, University College London, London, UK; 3 Institute for Life Course Health Research, Stellenbosch University, Stellenbosch, South Africa; 4 Department of Psychology, Institute of Psychiatry, Psychology & Neuroscience, King’s College London, London, UK; 5 Institute of Neurology, University College London, London, UK; 6 Young People’s Advisor Group, London, UK; 7 Parent and Carer Advisory Group, London, UK; 8 Centre for Family Research, Department of Psychology, University of Cambridge, Cambridge, UK

**Keywords:** Explore, Exploit, Psychosis, Depression, Anxiety, Transdiagnostic

## Abstract

In many everyday decisions, individuals choose between trialling something novel or something they know well. Deciding when to try a new option or stick with an option that is already known to you, known as the “explore/exploit” dilemma, is an important feature of cognition that characterises a range of decision-making contexts encountered by humans. Recent evidence has suggested preferences in explore/exploit biases are associated with psychopathology, although this has typically been examined within individual disorders. The current review examined whether explore/exploit decision-making represents a promising transdiagnostic target for psychosis, depression, and anxiety. A systematic search of academic databases was conducted, yielding a total of 29 studies. Studies examining psychosis were mostly consistent in showing that individuals with psychosis explored more compared with individuals without psychosis. The literature on anxiety and depression was more heterogenous; some studies found that anxiety and depression were associated with more exploration, whereas other studies demonstrated reduced exploration in anxiety and depression. However, examining a subset of studies that employed case-control methods, there was some evidence that both anxiety and depression also were associated with increased exploration. Due to the heterogeneity across the literature, we suggest that there is insufficient evidence to conclude whether explore/exploit decision-making is a transdiagnostic target for psychosis, depression, and anxiety. However, alongside our advisory groups of lived experience advisors, we suggest that this context of decision-making is a promising candidate that merits further investigation using well-powered, longitudinal designs. Such work also should examine whether biases in explore/exploit choices are amenable to intervention.

**Supplementary Information:**

The online version contains supplementary material available at10.3758/s13415-024-01186-9.

## Introduction

In many everyday decisions, individuals choose between trialling something novel or sticking with something they know well. Do you try out the new pizzeria that has opened across the road or go to your favourite café for lunch? Deciding when to try a new option or stick with an option that is already known to you, known as the “explore/exploit” dilemma, is an important feature of cognition and is present across several decision-making domains (Gabay & Apps, 2021). Explore/exploit decision-making relies on a range of cognitive faculties, such as motivational drives and executive functions (Lloyd et al., 2023). Notably, the cognitive faculties responsible for explore/exploit decision-making are also implicated in the onset and maintenance of psychopathology, including psychosis, depression, and anxiety (Addicott et al., 2017). It is therefore plausible that explore/exploit decision-making may be a transdiagnostic risk factor for psychopathology and one that has the potential to significantly improve life outcomes if targeted for intervention (Lloyd et al., 2023). This view is shared by those with lived experience of psychosis, depression, and anxiety, who have stated the topic is one that affects their everyday lives as they “*have a negative bias [towards] trying new things*” or conversely might try to “*escape the feelings that come with the illness*” by exploring new, potentially harmful alternatives (e.g., drugs and alcohol). In this paper, we systematically review whether explore/exploit decision-making as a potential transdiagnostic target for psychosis, depression, and anxiety.

Explore/exploit decision-making requires the individual to choose between exploiting an option with a known reward value and exploring a novel option with a less known or unknown reward value (Costa et al., 2019; Lloyd et al., 2023). Accounts of optimal explore/exploit decision-making suggest that individuals should engage in some level of both exploration and exploitation, and biases toward either option are thought to lead to suboptimal outcomes (Speers & Bilkey, 2023). For example, too little exploration can mean individuals remain with suboptimal options and do not discover other potentially better options, whereas too much exploration can mean individuals do not remain with a single option long enough to exploit the available rewards. Moreover, it has recently been proposed that there are normative changes to exploration across the lifespan, such that younger individuals should explore more compared with older individuals (Lloyd et al., 2023). These accounts suggest optimal explore/exploit decision-making involves a balance between both choices and individuals may exhibit biases in this context of decision-making by relying too much on one strategy alone.

A range of behavioural paradigms have been used to measure explore/exploit decision-making, including n-armed bandit (Daw et al., 2006), horizon tasks (Wilson et al., 2014), and patch foraging (Lloyd et al., 2021) (see Text Box 1). These tasks provide particularly rich behavioural measures, because they also are amenable to computational modelling (Daw et al., 2006)—a method that allows for exploration to be precisely quantified using mathematical formulas (Wilson & Collins, 2019). Explore/exploit tasks and their associated computational models provide a rich, ecologically valid source of information about decision-making (Speers & Bilkey, 2023) and can yield unique insights into the cognitive biases associated with psychopathology (Addicott et al., 2017).

Early computational models of explore/exploit choices measured exploration using an inverse temperature parameter. This parameter measures the frequency with which the individual selects the option that, to the best of their knowledge, does not yield the highest number of rewards (Wilson & Collins, 2019). While the stochasticity parameter can measure exploration, it is limited insofar as it can also reflect participants’ inattention to the task (Zorowitz et al., 2023). To address the limitation of these models, several new models have since been developed that better distinguish exploration from random responding (Costa et al., 2019; Daw et al., 2006; Gershman, 2019). These models also measure different types of exploration. For example, strategic forms of exploration where the individual receives a subjective bonus from exploring novel options to reduce uncertainty in their environment (Gershman, 2019). In addition, novel tasks have been developed that better parse different types of exploration. For example, Horizon tasks differentiate between exploration used to gain knowledge about the environment that has utility for future choices, known as directed exploration, and more stochastic forms of exploration without utility for future decisions, known as random exploration (Wilson et al., 2014). Given the variation in which exploration can be measured across different tasks and computational models, it is important to consider the methods used to measure biases in explore/exploit choices in relation to psychopathology.

Text Box 1: Tasks used to measure explore/exploit decision-making.

**Table:** 

n-armed Bandit: Participants are presented with two or more “bandits” or slot machines, which each have a probability or yielding a reward. Some bandits will have a higher probability of yielding a reward and participants must sample the available options to determine which bandit has the highest reward yield. Exploration on these tasks is quantified as the number of times participants select the bandit with the lower probability of yielding a reward or is formally quantified using computational modelling.
Reversal learning tasks: Like n-armed bandit tasks, reversal learning tasks present participants with two or more stimuli with probabilistic reward schedules. Participants select between these options, with one having a higher likelihood of yielding a reward. The probabilities associated with these options changes after a set number of trials and participants are required to update their estimate of the option expected to yield the highest reward. Exploration on these tasks is operationalised as the number of times a participant selects the option with the lower probability of yielding a reward or is formally quantified using computational modelling.
Horizon tasks: This task requires the participant to select between two slot machines that yield a varying number of points. Some points are displayed, and others are obscured. However, the task is manipulated such that one slot machine has a higher overall reward yield than the other. There are two conditions in this task, which manipulate the number of times the participant can sample each slot machine to accumulate points. In the short horizon manipulation, participants have fewer opportunities to sample machines to collect points, whereas in the long horizon manipulation participants have several attempts at sampling machines. The Horizon task measures two types of exploration: Directed exploration (i.e., sampling the lower valued machine in the long horizon condition) and random exploration (i.e., sampling the lower valued machine in the short horizon condition).
Patch foraging: Patch foraging tasks require the decision-maker to choose between a patch with gradually depleting rewards or exploring a new patch with a fresh distribution of rewards. Exploration incurs a time cost and therefore the participant must determine the optimal point to leave their current patch to explore a novel one. Exploration on these tasks is quantified as the threshold of rewards the participant selects before each leaving decision.

Explore/exploit decision-making relies on a complex set of neural circuits, many of which have been associated with psychopathology. Determining whether to exploit a known option or explore a novel one requires the individual to evaluate the value of staying with a known option or exploring a novel option. The explore/exploit trade-off therefore relies on reward processing abilities and neuroimaging work has suggested the dopaminergic reward system is responsible for determining the value of available options (Costa et al., 2019; Hogeveen et al., 2022). That the dopaminergic reward system has been implicated in explore/exploit choices is notable, as psychosis has been associated with hyperactivity in the dopaminergic reward system (Kapur et al., 2005; Kesby et al., 2018). In contrast, there is evidence of hypoactivity in the dopaminergic reward system in individuals with depression and anxiety compared with those without these diagnoses (Dunlop & Nemeroff, 2007; Nestler & Carlezon, 2006). In addition, studies have identified the anterior cingulate cortex, amygdala, and medial prefrontal network as regions that monitor the value of other options available to explore (Daw et al., 2006; Kolling et al., 2012). Notably, interactions between these regions have been associated with psychopathology (Park et al., 2018). Together, these studies suggest that there are significant overlap in networks responsible for explore/exploit decision-making and those associated with psychopathology.

Research in nonhuman animals has provided further insight into the contribution of specific networks of neurons to explore/exploit strategies. For example, in rhesus macaques, activation in the amygdala and ventral striatum are associated with encoding the value of exploration and exploitation choices (Costa et al., 2019). Furthermore, recent work in mice has identified a series of cells within the median raphe nucleus, a region associated with the promotion of anxiety-like behaviours (Abela et al., 2020), in promoting exploratory and exploitative choices (Ahmadlou et al., 2023). Work in human and nonhuman animals suggests several common neural networks involved in explore/exploit decision-making that may provide insight into the association between explore/exploit biases and psychopathology.

Considering common neural mechanisms underlying explore/exploit biases in those with psychosis, depression, and anxiety is pertinent in the context of transdiagnostic approaches to mental health. Derived from observations of heterogeneity within mental health classifications (Dalgleish et al., 2020) and comorbidity across diagnoses (Eaton et al., 2023; McGrath et al., 2020), transdiagnostic approaches are designed to identify common risk factors for mental health problems beyond traditional diagnostic categories (Krueger & Eaton, 2015). According to transdiagnostic approaches, an individual’s likelihood of experiencing mental health problems is predicted by a general risk factor for psychopathology (the “p-factor”; Caspi & Moffitt, 2018). Consistent with transdiagnostic approaches, biases in explore/exploit choices are not unique to any single diagnosis but occur across a range of mental health problems including affective disorders like anxiety (Fan et al., 2023) and depression (Blanco et al., 2013) and are present in individuals with psychosis (Strauss et al., 2011). This evidence could indicate that explore/exploit biases are associated with an individual’s general risk factor for psychopathology (i.e., their p-factor). To determine whether explore/explore choices are a viable transdiagnostic target, the current review will examine biases in explore/exploit decision-making across psychosis, depression, and anxiety.

Examining biases in explore/exploit decision-making can provide novel insight into behaviours commonly observed in those with anxiety, depression, and psychosis (Brown et al., 2023; Speers & Bilkey, 2023). For example, biases to engage in greater levels of exploration and less exploitation may reflect assumption that one’s surroundings are unstable/changeable in those with psychosis (Charlton et al., 2022) and increased avoidance of uncertainty in those with anxiety (O’Briein et al., 2019; Zorowitz et al., 2020). Specifically, exploring available options can reduce the uncertainty in one’s environment and may be used by individuals with anxiety to reduce uncertainty about their surroundings (Morris et al., 2016). In contrast, biases to exploit only what is known to the individual may reflect reduced pleasure in new experiences reported by those with depression (i.e., anhedonia; Pizzagalli, 2014; Watson et al., 2020) and behavioural inhibition arising from feelings of worry or unease reported by individuals with anxiety (Muris et al., 2001). Through understanding the link between biases in explore/exploit choices and the symptomology of psychosis, depression, and anxiety, future work can longitudinally investigate whether explore/exploit preference predict, or result from, changes to mental health.

Indeed, determining whether biases in explore/exploit choices are a cause or effect of mental health problems is important, because biases in this behaviour can have significant negative impacts on those with psychosis, depression, or anxiety. Our lived experience advisors expressed that biases to exploit can mean that individuals “*don’t see the positive sides of those new opportunities and stick with what they know instead of exploring new experiences”* but that biases for exploration also may have “*negative impacts if the activities are negative,*” such as trialling illicit drugs. These reflections suggest that biases in explore/exploit decision-making may compound existing mental health problems by reinforcing behaviours that exacerbate mental health problems. For example, exploiting known opportunities may lead individuals to withdraw from new opportunities, increasing negative affect (Heller et al., 2020), whereas exploring new, risky opportunities may expose individuals to experiences that can increase the likelihood of experiencing mental health problems (e.g., substance use; Lipari et al., 2016). Therefore, examining biases in explore/exploit choices can provide unique insight into the interaction between biased decision-making and the onset and maintenance of psychopathology.

Biases in explore/exploit decision-making may be driven by the cognitive faculties that are impacted by these diagnoses. For example, psychosis has been associated with a bias to perceive one’s surroundings as changeable (i.e., volatile; Cole et al., 2020). In volatile environments, the options expected to yield the greatest reward are changeable; therefore, the decision-maker should explore more often to uncover these changing reward associations (Behrens et al., 2007; Browning et al., 2015). Because psychosis has been associated with a bias to perceive one’s surroundings as volatile (Cole et al., 2020), individuals with psychosis may explore more than individuals without psychosis. In contrast, depression has been associated with reduced motivational drives that affect goal-directed behaviour (Grahek et al., 2019). Exploring novel alternatives rather that remaining with a known option requires the recruitment of motivational resources (Chong et al., 2017), which could suggest that depression will be associated with reduced exploration. Finally, anxiety has been associated with intolerance to uncertainty (Carleton et al., 2012; Osmanağaoğlu et al., 2018). One method to reducing uncertainty about one’s environment is to sample the available options (i.e., engaging in directed exploration), rather than remaining with a single known option (Gershman, 2019), indicating anxiety could be associated with increased exploration (however see Krypotos et al., 2022). Through understanding the link between biases in explore/exploit choices and cognitive faculties associated with psychosis, depression, and anxiety, future work can longitudinally investigate whether explore/exploit preference predict, or result from, changes to mental health.

The current review examines whether psychosis, depression, and anxiety are associated with biases in explore/exploit choices. Based on previous work (Brown et al., 2023; Furl et al., 2022; Pizzagalli, 2014) and the views of our lived experience advisors, we preregistered predictions (https://osf.io/tmcxz) that a) participants with psychosis will explore more than participants without psychosis, b) participants with depression/depressive symptoms will explore less than participants without depression, and c) participants with anxiety/anxiety symptoms will explore more than participants without anxiety.

## Methods

### Lived experience advisory groups

We recruited two advisory groups of lived experience experts through an existing network of advisors engaged in another project, and through social media advertisement. The first advisory group was a young people’s advisory group comprised of five individuals (aged between 16–24 years, 100% female) with lived experience of psychosis, depression, or anxiety based across Nigeria, South Africa, and the UK. The second advisory group was comprised of two parents and carers of young people with psychosis, depression, or anxiety based in the United States (100% female). Applicants were asked to provide a short, written application detailing their lived experience relevant to the topic. However, we did not require participants (or in the case of the Parent and Carer Advisory Group, their child) to have a formal diagnosis to be considered eligible for the advisory groups and therefore did not collect any questionnaire data from these individuals. Each group met four times and were involved in priority setting, interpreting the results, and supporting with dissemination activities (see Supplementary Materials). Lived experience advisors were remunerated £20 per hour for their role. As lived experience advisors do not qualify as research participants, the involvement of these individuals did not require approval from an ethical review board (Co-Production Collective, 2023).

### Eligibility criteria

Studies were considered eligible if they met the following criteria:

1. Human participants (adults, adolescents and children).
2. Clinically diagnosed participants or participants exhibiting elevated levels of psychosis, depression or anxiety.
3. Measured explore/exploit decision-making using an established task.
4. Reported at least one metric of exploration behaviour (e.g., use of directed versus random exploration, frequency of exploration, inverse temperature parameter, etc.).
5.  Included an explicit reinforcer (i.e., rewards or punishment).

### Search strategy

**Fig. 1:**
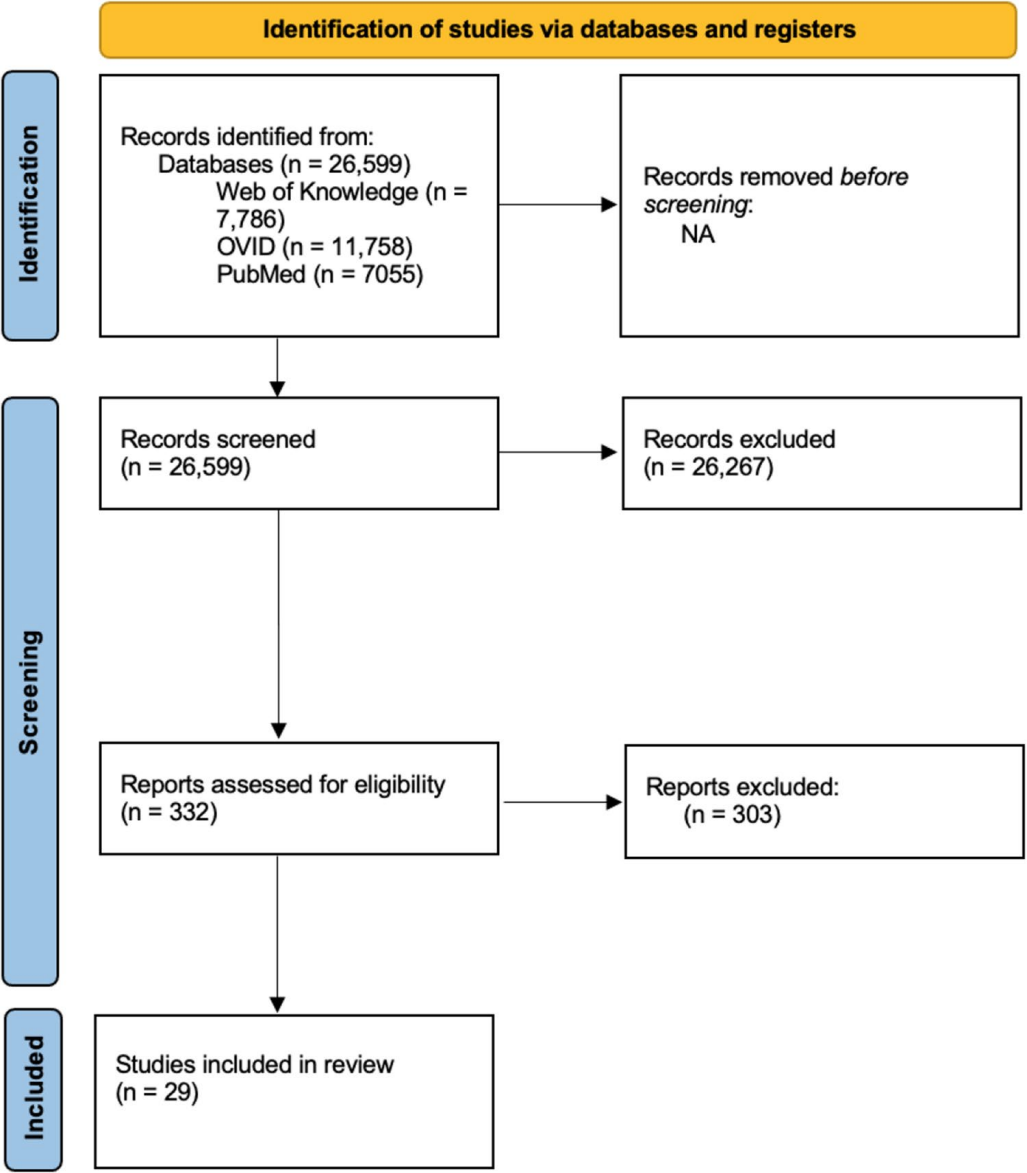
PRISMA flow chart highlighting the number of records identified from databases

We preregistered our search strategy on the Open Science Framework (https://osf.io/tmcxz). Searches were conducted between April–May 2023. EMBASE, MEDLINE, PsychINFO, and Web of Knowledge data were searched for eligible studies. The term used to search databases was: (phobia OR anxi* OR psycho* OR schizo* OR depressi* OR major depressive disorder OR bipolar disorder OR OCD) AND (explore-exploit OR bandit OR forag* OR probabilistic reversal OR reversal learning) (Fig. 1). Included studies were screened for bias by using the Appraisal tool for Cross-Sectional Studies (AXIS; Downes et al., 2016).

### Study selection and data extraction

The full text of studies identified from the databases were independently screened by two reviewers (AB & ZF) against the eligibility criteria. Any disagreements about inclusion were discussed between AB, ZF, and AL and a consensus reached (see https://osf.io/tmcxz). The following data from eligible studies were then extracted into a Microsoft Excel file:

- Study characteristics: Study authors, year of publication, country study was conducted, study design, recruitment method, allocation method (if applicable).
- Participant characteristics: Inclusion/exclusion criteria, follow-up period (if applicable), sample size, age (sample mean and SD), gender (% male, female, or other), ethnicity, socioeconomic information, diagnosis or symptoms measure, method used to measure diagnosis or symptoms, comorbid diagnoses, or symptoms.
- Task characteristics: Description of explore/exploit task, number of trials, reinforcer, remuneration for participation, explore-exploit measure(s), analysis method.
- Outcome information: Main results, missing data.
- Potential moderators: Gender distribution in the sample, age, concurrent pharmacological treatments, concurrent therapeutic treatments.
- General information: Limitations identified by the study author(s), funding information.

### Data synthesis

Because of the heterogeneity across studies, including the populations recruited, diagnosis method, and explore/exploit tasks used, we were unable to conduct a meta-analysis or any quantitative synthesis. Rather, we synthesised the findings as a narrative review. To avoid any conflicts of interest, literature searches, data extraction, and risk of bias assessments were conducted by members of the research team who had not been involved in any of the studies identified.

## Results

### Summary of studies

Twenty-nine studies met our inclusion criteria, which included a total of 4945 participants. The mean age of participants across all included studies was 34.02 (range 20.50–70), and the percentage of female participants was 47.35% (see Table 1 for demographic information).

**Table 1:** Demographic inf﻿ormation

First author	Reference	Country	Recruitment method	Inclusion/exclusion criteria	Sample size	Mean age (SD)	Gender (% female)	Ethnicity	Diagnostic method	Comorbidities	Explore/exploit task	Explore/exploit measure	Result	Potential moderators
Aberg	(Aberg et al., 2022)	Israel	NR	Right handed, no previous history of psychiatric or neurological disorders	Behavioural: 20, fMRI: 28	Behavioural: 26.70 (0.97). fMRI: 27.57 (0.73)	Behavioural: 70% fMRI: 71.43	NR	**Anx:** Spielberg’er TAI (self-report)	NR	3-armed bandit	Proportion of exploratory choices, directed and random exploration	Exploration positively correlated with anxiety (F(1,26) = 24.98, *p* < 0.001, η2 *p* = 0.490)	NA
Aylward	(Aylward et al., 2019)	UK	Advertising campaigns in local. Healthy Controls from institution	Unmedicated mood and anxiety symptoms	132, 88 healthy controls and 44 with unmedicated mood and anxiety	Healthy controls 23 (5.1), Unmedicated mood/anxiety 28 (9.8)	HC : 56.82% Unmedicated: 63.64%	NR	**Anx** and **Dep**: Mini International Neuropsychiatric Interview	Panic disorder	4-armed bandit under threat of shock and safety conditions	Lapse parameter	Positive correlations between anxiety, depression and lapse parameter *r*(130) = 0.32, *p* < 0.001	NA
Brolsma	(Brolsma et al., 2022)	Netherlands	Outpatient group contacted, control group contacted from databases of previous studies and external advertisement	Inclusion: Current depressive disorder, anxiety disorder, addictive disorder, ADHD, or ASD were eligible. Exclusion: Current psychotic disorder, IQ <70, sensorimotor disorder, not mentally competent to consent, or insufficient knowledge of Dutch language.	No MDD group 61, remitted MDD 55, current MDD 191, HC 81	No MDD 40.6 (14.6), remitted MDD 37.5 (13), current MDD 41.5 (14.8), healthy controls 40.3 (17)	No MDD: 31.15% Remitted MDD: 45.45% Current MDD: 38.61% Healthy Controls: 49.38%	NR	**Dep**: Inventory of Depressive Sympommology (self-report). **Anx**: Anxiety severity index	ADHD, Autism, anxiety, addictive disorder	Probabilistic reversal learning task	Computational modelling of inverse tempreature	Decision variability did not differ between the four groups (F(3,285) = 2.16, *p* = .093 η^2^_p_ = 0.022	NA
Bustamante	(Bustamante et al., 2022)	USA	Prolific	Failure to complete attention check.	678	24.5 years (6.7)	71.68%	NR	**Dep**: PHQ-9; Apathy Motivation Index. **Anx:** GAD-7	NR	Effort patch foraging	Point at which participant chose to leave their current patch to explore a novel one.	Decreased cognitive effort cost associated with decreased anxiety. Decreased cognitive effort cost negatively associated with anhedonia and depression.	NA
Cathomas	(Cathomas et al., 2021)	Switzerland	Patients with schizophrenia recruited from inpatient and outpatient units. HC recruited using adverts	Exclusion: medication changes in the previous 2 weeks, not clinically stable, any diagnoses other than schizophrenia, use of benzodiazepines except lorazepam >1 mg per day, lifetime alcohol use disorder, current cannabis abuse or dependency, other substance abuse, history of head injury, any autoimmune or chronic inflammatory disorder, any use of pain medication or anti-inflammatory drugs in at least 1 week prior to testing, any known acute inflammation 2 weeks prior to testing, or acute psychosis symptoms	HC 19, schizophrenia patients 45	HC 32.53 (9.45), schizophrenia patients 34.00 (10.47)	HC: 52% SZ: 31%	NR	**Psy**: Mini-International Neuropsychiatric Interview, PANSS, (researcher administered)	NR	3-armed bandit	Directed and random exploration based on uncertainty associated with each option	Exploitation was reduced in patients with SZ (t = 2.14, *p* = 0.036, d = 0.5. SZ patients showed more random exploration (U = 275.5, *p* = 0.042, d = 0.65), but similar levels of directed exploration (U = 302, *p* = 0.104).	Concurrent pharmacological treatments and multimodal therapy program
Deserno	(Deserno et al., 2020)	Germany	NR	Medicated with diagnosis of schizophrenia or no diagnosis of schizophrenia	HC 43, SZ 46	HC 34.40 (8.24), SZ 35.07 (7.596)	HC 14 (30.4%), SZ 13 (30.2%)	NR	Mini-International Neuropsychiatric Interview, PANSS, (researcher administered)	NR	Reversal learning	Switching behaviour and inverse temperature parameter	SZ switched more, independently of feedback from the previous trial (main effect group F = 27.77, *p* < 0.001, feedback x group F = 0.02, *p* = 0.89).	Standardised chlorpromazine treatment.
Dezfouli	(Dezfouli et al., 2019)	Australia	Outpatient mental health clinics and from the surrounding community	Exclusion: History of neurological disease, medical illness known to impact cognitive and brain function, intellectual and/or developmental disability and insufficient English for neuropsychological assessment.	HC 34, unipolar depression 34, bipolar 33	HC 23.6 (4.3), unipolar depression 21.6 (2.5), bipolar 23.1 (4.4)	HC 19 (55.8%), unipolar depression 19 (55.8%), bipolar 24 (72.7%)	NR	**Dep**: Hamilton Depression Rating Scale (researcher administered)	Mania (bipolar)	Probabilistic learning task featuring binary choice	Computational model with inverse temperature parameter	Only descriptive information reported for model parameters. Depressed participants had lower inverse temperature	Concurrent pharmacological treatments
Dombrovski	(Dombrovski et al., 2010)	USA	Inpatient or outpatient treatment at Pittsburgh hospital	Diagnosed MDD without psychotic features, score ≥24 on the Mini-Mental State Examination. Exclusion: sensory disorders that precluded cognitive testing, delirium, neurologic disorders, bipolar disorder, schizophrenia, schizoaffective disorder, and exposure to ECT in the previous 6 months were excluded.	65: 15 depressed suicide attempters, 12 depressed suicide ideators, 24 nonsuicidal depressed elderly, and 14 nondepressed nonsuicidal elderly.	Nondepressed 65.6 (4.9), non-suicidal depressed 70.0 (7.4), suicide ideators 68.8 (5.6), suicide attempters 66.8 (7.8)	ND: 2 female (14%), non-suicidal dep 13 female (54%), suicide idea 5 female (42%), suicide attempt 9 female (60%)	ND 13 white (93%), non-suicidal dep 20 white (83%), suicide ideators 11 (83%), suicide attempters 11 white (73%)	**Dep**: Structured clinical interview	Suicidal Ideation	Probabilistic reversal learning task	Computational model measuring inverse tempreature	Exploration parameter did not vary between groups (F(3,61) = 0.59, *p* = 0.62)	Concurrent pharmacological treatments
Fan	(Fan et al., 2023)	USA	Prolific.	Based in USA, completed at least 10 tasks but fewer than 1000 tasks on Prolific Exclusion: age outside the range of 18-65, chose the more rewarding option < 60% of trials in the two-armed bandit task, did not complete anxiety-related questionnaires.	Experiment 1: N = 531; Experiment 2: N = 576	Exp 1: 36.1 (10.9), exp 2: 35.3 (10.5)	Exp 1: 219 female (43.71%), exp 2: (40.7%)	NR	**Anx**: STAI, STCSA, (self-reported)	Depression	2-armed bandit	Directed and random exploration	People high on trait somatic anxiety were less likely to engage in directed exploration (t(150,273) = −2.12, *P* = 0.034, β = −0.070). Somatic anxiety associated with increased sensitivity to value difference between options (t(150,273) = 3.32, *p* < 0.001, β = 0.217)	NR
Harlé	(Harlé et al., 2017)	USA	Advertised on University of California San Diego online participant sign up system	Minimal depression severity (i.e., BDI-II score > 8), good general health, sufficient proficiency in English Exclusion criteria: lifetime history of psychotic, bipolar or obsessive-compulsive disorder, history of current alcohol or substance dependence, recent history of (i.e., within last 6 months) or currently taking any antidepressant or psychotropic medications (except occasional sleep aid)	53	20.5	38 female (71%)	NR	**Dep:** Beck Depression Inventory. **Anx**: State Anxiety Inventory (self-report)	NR	2-armed bandit	Computational model with an inverse temperature parameter	Negative association between anhedonia and inverse tempreature parameter (r = −0.52, *p* = .004).	Age
Kaske	(Kaske et al., 2023)	USA	Flyers and emails.	Age >18 years, hair length >3 cm, occupation in health care, and residency	123 completed survey, 66 completed task	Of survey completers, m = 34, range 20–61	105 (85.36%)	American Indian 1 (0.8%), Asian 15 (12.2%), Black 0 (0%), Native Hawaiian 1 (0.8%), Other 6 (4.88%), White 100 (81.3%)	**Dep**: PHQ-9. **Anx**: GAD-7 (self-report)	NA	2-armed bandit	Computational model measuring inverse temperature	High levels of depressive symptoms interacted with cortisol levels to negatively predict exploration (β = −0.49, pFDR = .022	NA
Kattagen	(Katthagen et al., 2022)	Germany	Schizophrenia patients from inpatient and outpatient units	Inclusion: free of medication for at least 5 half-lives of previous antipsychotic treatment. Controls: no past or present Axis I disorder Exclusion criteria: no current or past history of drug dependence	20 SZ and 23 HC	SZ: 33.2 (9.5), HC: 32.2 (8.2)	SZ 7 females (36.84%), HC 7 (30.4%)	NR	**Psy:** ICD-10 (researcher administered)	NR	Probabilistic reinforcement task	Inverse temperature parameter	SZ patients had lower inverse temperature parameter compared with HC (t = 3.0, *p* = .004).	Gender
Martinelli	(Martinelli et al., 2018)	UK	NR	Inclusion: capacity to consent, age between 18–60 years, English proficiency and IQ > 80. Exclusion: current drug or alcohol dependence, brain disease or damage, used psychotropic medication (except patients).	24 SZ, 24 HCs	SZ 41.50 (6.76), HC 40.50 (7.58)	SZ 3 female (15%), HC 3 female (12.5%)	NR	**Psy**: ICD-10 (Clinician reported)	NR	3-armed bandit	Computational modelling with an inverse temperature parameter	No statistically significant difference between individuals with or without Schizophrenia on the inverse temperature parameter (T(42) = 1.897, *p* = .065, d = 0.57)	Gender and concurrent pharmacological treatments
Mukherjee	(Mukherjee et al., 2020)	USA	Flyers and referral.	Inclusion: diagnostic criteria for current MDD episode, had no history of substance abuse/dependence in the past 6 months, and had no history of bipolar disorder and/or psychotic episodes. Controls: No history of psychiatric illness and no psychotropic medicine.	64 MDD – 64 HC	MDD group - 40.45 (13.48) Control - 38.53 (11.73)	MDD 3 female (15%), HC 3 female (12.5%)	MDD - Black or African American — 34 (53% White – 24 (38%),Asian – 4 (6%), Other – 2 (3%) Control- Black or African American – 33 (52% White – 23 (36%),Asian – 7 (11%), Other – 1 (2%),	**Dep**: Structured Clinical Interview D (clinician assessed)	Bipolar, substance abuse, psychotic disorders.	Reward Probabilistic reversal learning task with both reward and punishment conditions	Computational model with inverse temperature parameter	Differences between those with and without depression on inverse temperature parameter in punishment condition (*p* = .04).	Concurrent treatment and medication
Reddy	(Reddy et al., 2016)	USA	Patients were recruited from outpatient clinics, and from local clinics and housing facilities.	Schizophrenia diagnosis, aged between 18 – 60, no neurological disease, no serious head injury, no substance dependence or significant abuse in the previous month, no developmental or intellectual disability, clinically stable, no change in medication in prior 4 weeks.	SZ: 126 HC: 72	SZ: 48.8 (11..2)HC: 46.7 (8.1)	SZ– 40 female (31.7%) HC – 32 (44.4%)	NR	**Psy**: Clinical Assessment Interview (Clinician assessed)	NR	Probabilistic reversal learning task	Proportion of errors (i.e., selecting the lower value option)	Patients (*M* = 0.45; SD = 0.11) had a significantly higher proportion of error trials than controls (*M* = 0.40; SD = 0.11; *t* = 2.93, *p* < .01).	NR
Robinson	(Robinson et al., 2022)	Australia	Data was used from a larger cognitive data bank reported in previous studies.	Methamphetamine dependency, with use within 21 days but no use in the last 3 days. No drug dependencies other than alcohol, cigarettes and marijuana. No loss of consciousness longer than 30 minutes, psychosis disorder diagnosis, or intellectual disability.	106 Clinical and 48 control participants	Clinical- 31.20 (7.25), Control - 31.59 (8.67)	Clinical – 27 (25.4%) Control – 12 (74.5%)	NR	**Dep**: Centre for Epidemiological Studies Depression Scale self-report)	Methamphetamine use disorder	Iowa Gambling Task (IGT)	Computational model including an inverse temperature parameter	No association between depression and inverse temperature parameter (r = −0.24) or directed exploration bonus (r = 0.10).	NR
Schlagenhauf	(Schlagenhauf et al., 2014)	Germany	The schizophrenia patients were recruited from a clinical mental health service. Healthy participants were volunteers.	No other psychiatric axis I disorder. Healthy controls had no psychiatric disorder or family history of psychiatric disorders.	N = 48 (24 SZ, 24 HC)	SZ - 27.5 (SD 5.2, 21-40), HC - 27.2 (SD 4.9, 20 - 40)	SZ – 2 (8.3%) HC – 2 (8.3%)	NR	**Psy**: DSM-IV and ICD-10 (clinician assessed)	NR	Reversal learning task	Number of correct choices (i.e., highest value option selected)	Patient group selected the higher value option significantly less often compared to controls (*p*s < .003)	
Sheffield	(Sheffield et al., 2022)	USA	Participants recruited from Vanderbilt Medical center.	Exclusion: Major physical or neurologic illness, active substance use disorder, significant head injury and IQ of <79. Healthy controls: no relative with a psychiatric disorder.	N = 86 (HC 44, SZ - 42	HC - 30.1 (8.0), SZ - 27.4 (10.2)	HC 17 (38.6) SZ – 11 (26.2%)	Control: Black – 9 (20%), Other – 5 (11%), White – 30 (68%) Patient: Black – 12 (29%), Other – 3 (7%), White – 27 (64%)	**Psy**: Structured Clinical Interview (clinician assessed)	Anxiety and depression	3-armed probabilistic reversal learning task	Win switch rate, lose-stay decisions	Participants with schizophrenia exhibited more win switches (F1,84 = 9.21, *p* = .003, h2 = 0.10), and less lose stays (F1,84 = 4.53, *p* = .04, h2 = 0.05).	NR
Smith	(Smith et al., 2020)	USA	Identified from existing dataset	Exclusion: Positive drugs test, met criteria for psychotic, bipolar, or obsessive-compulsive disorders, or reported history of moderate-to-severe traumatic brain injury, neurological disorders, severe or unstable medical conditions, active suicidal intent or plan, or change in medication dose within 6 weeks.	N = 198 (Healthy control - 51, Clinical: 147; propensity matched - 49)	HC - 32.27 (11.35), Clinical - 34.06 (9.17) Propensity matched HC 30.35 (11.40), SUDS - 32.25 (7.72)	Sex HC male .44 (.50), Clinical - .49 (.50) Propensity matched - HC - .45 (.50), SUDS - .55 (.50)	NR	**Dep**: PHQ-9, **Anx**: OASIS (self report)	Substance use disorder	3-armed bandit task	Random exploration, goal directed exploration and insensitivity to information parameter	Negative correlations between reward sensitivity parameter and OASIS (r = −.19, *p* = .02). Participants with MDD had lower reward sensitivity than those without (t = 3.17, *p* = .002).	
Smith	(Smith et al., 2022)	USA	Convenience sample of University of Arizona students and surrounding community.	Minimimum age was 18 otherwise NR.	N = 416	23.75 (5.61)	301 (72.4%)	NR	**Dep**: Beck Depression Inventory, **Anx**: State Trait Anxiety Inventory (self-report)	NR	Horizon task	Directed and random exploration.	Directed exploration negative associated with state anxiety (b = −0.1, CI = [−0.17, -0.03], BF = 7.56	
Suetani	(Suetani et al., 2022)	Australia	Patients from clinical services. Controls via brochures.	Patient inclusion: Diagnosis of Persistent Psychotic Disorder. Between the ages of 18 and 50. HC inclusion: No diagnosis of a psychotic disorder and had not experienced a psychotic episode.	N = 79 (Psychotic disorder – 45, Healthy controls – 34)	Conrols – 32.4 (9.9), Patients – 31.0 (8.8)	Control – 65.9% Patients – 27.7%	NR	Psy: Clinical diagnosis	Substance abuse	Serial-reversal learning task	Computational model measuring the inverse temperature	Persistent psychosis subjects had lower inverse temperature (F1,76 = 8.6, *p* < 0.01).	NR
Takano	(Takano et al., 2019)	Belgium	Sample pool at Leuven University.	Fluent Dutch Speakers.	N = 46	23.1 (5.6)	37 (80.4%)	NR	**Dep**: Beck Depression Inventory (self-report)	NR	Probabilistic reversal learning task	Inverse temperature parameter	No association between depression measure and exploration parameter (r = 0.03).	Groups not matched for gender or age
Taylor	(Taylor et al., 2023)	USA	Previous data set.	Inclusion - PHQ-9 ≥ 10, OASIS ≥ 8, and/or Drug Abuse Screening Assessment (DAST-10 ≥ 3. Exclusion - Positive tests for drug use, meeting criteria for psychotic, bipolar, or OCD, a history of moderate-to-severe traumatic brain injury, neurological disorders, or severe or unstable medical conditions, active suicidal intent or plan, or change in medication dose within 6 weeks.	N = 267 (HC – 99, Clinical – 168)	HC=32.29 (11.08) Clincial= 33.75 (8.34)	HC – 60 (60.6%) Clinical – 105 (62.5%)	NR	**Anx**: OASIS, **Dep**: PHQ-9	Substance use disorder	3-armed bandit task	Computational model with reward sensitivity parameter	No association between exploration parameter and OASIS or PHQ.	NR
Vandendriessche	(Vandendriessche et al., 2023)	France	Clinical center	Inclusion: Major unipolar depression, 18–65 years old. Exclusion: Psychotic symptoms, chronic psychosis, a serious personality disorder, neurological or somatic disorders, neuroleptic treatments, electroconvulsive treatments, current substance use. Controls: No current or past psychiatric diagnosis and no psychotropic medications.	N = 56) (MDD – 30 Control – 26)	MDD - 36.5 (2.80), HC – 40.35 (2.09)	MDD – 30 (53.3%) HC – 26 (61.5%)	NR	**Dep**: Mini International Neuropsychiatric Interview (Clinician assessed)	Anxiety related disorder and substance abuse	Probabilistic learning task	Inverse temperature parameter.	No significant difference between groups on the choice temperature parameter (t(48) = 1.64, *p* = 0.11)	Psychotropic medications
Waltz	(Waltz & Gold, 2007)	USA	Psychiatric Research Centre. Healthy controls recruited from newspaper adverts	Patient group: Clinically stable (physician assessed), No changes in type or prescription amount of medication for previous 4 weeks. Controls: No Axis I or II disorder. No family or personal history of psychosis or psychiatric disorder, which may impair cognition or drug dependency.	N = 50 (Patients - 34 , HC – 26)	Patients – 45.01 (11.30) HC – 45.47 (8.40)	HC – 12 (46.2%) Patient – 11 (32.3%)	NR	**Psy**: Structured Clinical Interview (clinician assessed)	Depression	Probabilistic discrimination learning task	Number of error trials.	Interaction between the group (patient versus healthy control) and learning stage [initial discrimination versus reversal; F(1, 39) = 4.12; *p* < 0.05]. Post-hoc tests confirmed the presence a group difference in reversal error rates t(39) = 2.12, *p* < 0.05	NA
Blanco	(Blanco et al., 2013)	USA	Recruited from University of Texas undergraduates	NR	N = 133 (33 Depressive, 95 controls	NR	NR	NR	**Dep**: Center for Epidemiological Studies Depression Scale (self-report)	NR	Leapfrog task	Choosing the result with the "highest" value termed exploitation.	Depressed participant selected the higher value option slightly less than non-depressives (t(131) = 1.92, *p* = 0.057, d = 0.37). Depressive participants explored more compared to nondepressives (t(131) = 4.14, *p* < 0.001, d = 0.80)	NR
Murphy	(Murphy et al., 2003)	UK	Inpatient or outpatient treatment for patient condition, controls recruited through adverts.	Patients: no psychoactive substance abuse or steroids. No history of neurological or medical disorders affecting cognition. Controls: no history of psychiatric disorder. No neurological history or substance abuse. Mild to low BDI symptoms.	N = 50 (Patients 27, Control – 23)	Patients 38.9 (9.7), Controls 39.1 (10.8).	Patients – 14 (51.8%) Control – 12 (52.2%)	NR	**Dep**: DSM-IV criteria (clinician assessed)	Anxiety and spatial working memory	Probability reversal task	Maintenance failure score	Maintenance failure scores significantly differ by group (F(1,37)= 5.74, *p* < 0.05) due to higher scores in depressed patients relative to controls	NR
Tavares	(Taylor Tavares et al., 2008)	UK	Clinics of the NIH Clinical Centre and controls via community advertising.	Exclusion: Met criteria for alcohol or substance abuse within last year, had ever met criteria for alcohol or substance dependency other than nicotine, were exposed to psychotropic medication in the last three weeks, showed structural abnormalities, had a history of brain injury or neurological disease, IQ below 85 or currently pregnant.	N = 30 (Control – 15, MDD, 13, BD, 12)	Control – 33.9 (2.33), MDD - 38.3 (2.30), BD – 33.4 (3.16)	Control – 4 (26.7%) MDD – 3 (23.1%) BD – 3 (25%)	NR	**Dep**: Structured clinical interview (clinical interview)	Anxiety	Probabilistic reversal learning task	Spontaneous errors that could not be attributed to misleading feedback or rule reversal	No significant difference between groups on spontaneous errors (*p* > 0.1)	NR
Waltz	(Waltz et al., 2020)	USA	Patients were outpatients from outpatient clinics.	Patient group: Clinically stable (physician assessed), No changes in type or prescription amount of medication for previous four weeks. Controls: No Axis I or II disorder. No family or personal history of psychosis or psychiatric disorder	N = 141 (108 patients, 33 controls)	Patients – 37.0 (10.1) HC – 36.4 (10.4)	Patients – 36 (33.3%) HC – 11 (33.3%)	Patients: Caucasian – 53 (49%), African American – 43 (40%), Asian – 4 (4%) mixed/ other – 7 (6%) Control: Caucasian – 18 (55%), African American – 13 (39%), Asian – 0, mixed/ other – 2 (6%)	**Psy**: Structured clinical interview for DSM–IV	NR	Horizon task	Direct and random exploration.	Patients showed reduced directed exploration (F(1,139) = 8.83, *p* = 0.003). Patients did not differ in random exploration (F(1,139) = 0.70, *p* = 0.403)	Antipsychotic medications

NR = not reported; Anx = anxiety; Dep = depression; Psy: psychosis; MDD = major depressive disorder; HC = healthy control; SZ = schizophrenia patient

### Psychosis

Ten studies examined differences between individuals with a diagnosis of psychosis and healthy controls on explore/exploit choices. Eight (80%) found that people with psychosis explored more compared with people without psychosis, one (10%) study did not detect a difference between people with and without psychosis on explore/exploit choices, and one study (10%) found people with psychosis explored less than people without psychosis. Most of the studies that examined explore/exploit biases in psychosis justified their samples sizes, suggesting they were sufficiently powered to detect a difference between patient and healthy controls groups. Based on these findings, we suggest that there is moderate-strong evidence for a bias toward greater exploration in people with psychosis, relative to those without psychosis.

The conflicting findings from two studies about the association between psychosis and explore/exploit choices may be explained by differences in methodologies and populations utilised by these two studies. The one study finding no difference between those with and without psychosis on explore/exploit choices reported that all participants were being treated with antipsychotic medication at the time of completing the explore/exploit task (Martinelli et al., 2018), the side effects of which can reduce exploration (Speers & Bilkey, 2023). Moreover, the authors suggest that the difference between their clinical and healthy control groups were trending towards significance, such that individuals with psychosis explored more than individuals without psychosis (Martinelli et al., 2018). In contrast, the eight studies finding greater exploration in those with psychosis reported variable levels of antipsychotic usage in their clinical populations. Furthermore, the one study that found less exploration in those with psychosis utilised a Horizon task (Waltz et al., 2020), specifically finding those with psychosis exhibited less directed exploration, whereas there was no difference between participants with and without psychosis on random exploration. In contrast to Waltz et al. (2020), the eight studies finding greater exploration in psychosis utilised reversal learning tasks. Horizon tasks measure directed and random exploration separately, whereas these two types of exploration are not disentangled in reversal learning tasks (however, see Speekenbrink & Konstantinidis, 2015), suggesting the association between psychosis and exploration may depend on the type of exploration being measured. Together, we suggest that there is moderate-strong evidence for heightened exploration in psychosis and that conflicting findings in the literature can be explained by differences in methodology or populations between studies.

### Depression

Seventeen studies examined the association between depression and explore/exploit choices. Unlike studies examining psychosis, there was significant heterogeneity in the samples, operationalisation of explore/exploit choices, and measurement of psychopathology in this literature. There also was significant heterogeneity in the association between depression and explore/exploit choices in these studies: Six studies (35.29%) found that participants with depression explored more than participants without depression, four (23.53%) studies found that participants with depression explored less than those without depression, and seven (41.18%) studies did not detect any association between depression and explore/exploit choices.

We preregistered that we would focus on studies recruiting participants with clinical levels of depression should enough studies be identified through our literature search (see “Miscellaneous search strategy details”: https://osf.io/tmcxz). In line with this preregistered methodology, we focussed on the ten (of a total of 17) studies that compared those with clinical levels of depression to a healthy control group. Studies that recruited participants with clinical levels of depression typically employed case-control methods, where participants are matched on age and sex, reducing the possibility of these variables confounding the findings. Notably, 60% of these ten studies also did not detect differences between clinical and healthy samples on measures that could confound task performance, such as IQ and Education (Brosolma et al., 2022; Dezfouli et al., 2019; Mukherjee et al., 2020; Murphy et al., 2003; Taylor Tavares et al., 2008; Vandendriessche et al., 2023). However, 30% of studies did not report this information (Aylward et al., 2019; Dombrovski et al., 2010; Harlé et al., 2017) and one study (10%) did identify differences between their clinical and healthy control groups (Robinson et al., 2022). Restricting the results to the ten studies that compared those with clinical levels of depression to healthy controls, six (60%) found that people with depression explored more than people without depression, whereas four (40%) did not detect an association between depression and explore/exploit choices.

Examining these studies in further detail, three of the studies that found no difference between participants with and without depression had a relatively low sample size (i.e., 30 or fewer participants per group), which could suggest they were underpowered to detect a difference between participants with and without depression (Dombrovski et al., 2010; Taylor Tavares et al., 2008; Vandendriessche et al., 2023). Furthermore, one of these studies recruited older adults only, with a mean age older than 60 years (Dombrovski et al., 2010). Empirical and theoretical work has suggested that exploration declines into older adulthood (Lloyd et al., 2023; Mata et al., 2013); therefore, the age of the sample in Dombrovski et al. (2010) may be a confounding variable in this study.

The heterogeneity in this literature was not informed by examining model fitting methods. While hierarchical estimation methods provide more accurate measures of parameters within groups (i.e., between clinical and healthy control groups; Mukherjee et al., 2020), there was not evidence that the model fitting method affected whether differences were detected between clinical and healthy control groups. Based on the available evidence, we cannot currently conclude whether depression is associated with biases in explore/exploit choices.

### Anxiety

Six studies investigated the association between anxiety and explore/exploit choices. Three studies (50%) found that anxiety was positively associated with exploration, whereas a further three studies (50%) found a negative association between anxiety and exploration. It is important to note that there was significant heterogeneity in the operationalisation of exploration in this literature. The three studies utilising n-armed bandit tasks consistently evidenced a positive association between anxiety and exploration, and scored “Good” on our AXIS measure. Examining the models used to measure exploration in n-armed bandit tasks, there was evidence that exploration was used as a strategy to reduce uncertainty about the environment in those with anxiety, rather than arising from a stochastic switching policy or inattention (Aberg et al., 2022; Smith et al., 2020).

The three studies demonstrating a negative association between anxiety symptoms and exploration used an alternative set of paradigms (specifically foraging, Horizon, or volatile bandit tasks). When directed and random exploration were measured using Horizon tasks, anxiety was associated with less directed exploration (Fan et al., 2023; Smith et al., 2022). Notably, individuals with heightened anxiety were found to underestimate uncertainty in the Horizon task (Fan et al., 2023). This finding could indicate that individuals with anxiety underestimate the uncertainty in Horizon tasks and therefore do not explore to reduce uncertainty in the task. In contrast, individuals with and without anxiety may perceive similar levels of uncertainty in n-armed bandit tasks. Under these conditions, our review suggests that individuals with anxiety explore more in these tasks to reduce the perceived uncertainty in their environment.

Sample characteristics may also contribute to the conflicting findings in the literature examining anxiety. Two of the three studies finding a negative association between anxiety and exploration recruited their sample from Prolific (Bustamante et al., 2022; Fan et al., 2023). This design feature is notable, as the levels of anxiety symptomology is lower in community samples than in clinical samples. These studies did not report the proportion of individuals who met the clinical cut off for anxiety; therefore, we cannot determine whether these studies had enough individuals who met clinical criteria for anxiety to study the association between clinical levels of anxiety and explore/exploit choices. Another study finding a negative association between anxiety and exploration recruited a convenience sample of predominantly female undergraduates (Smith et al., 2022), which may confound the association between anxiety and explore/exploit choices, as previous work has demonstrated sex differences in exploration choices (Bach et al., 2020; Chen et al., 2021). Compared with studies that employed case-controlled designs, studies recruiting community samples were less diverse in symptom severity and were overrepresented by female participants, which may obscure the true association between anxiety and explore/exploit choices.

### Is explore/exploit decision-making a transdiagnostic target?

The purpose of this review was to consider whether explore/exploit decision-making is a transdiagnostic predictor or psychosis, depression, and anxiety. Considering the results across the diagnostic categories reviewed, the findings of the included studies could be consistent with transdiagnostic approaches insofar as the association between explore/exploit biases and psychopathology was heterogenous within each diagnostic category. Moreover, there was a degree of homogeneity across diagnostic categories insofar as more severe presentations of psychopathology (i.e., those studies that recruited clinical populations) were associated with greater exploration compared to healthy controls.

To further understand whether explore/exploit decision-making is a promising transdiagnostic target, we examined whether specific symptom profiles were associated with biases across the included studies, where these were reported. Examining positive, negative, and disorganisation symptoms in psychosis, two studies (Cathomas et al., 2021; Reddy et al., 2016) found symptoms of disorganisation, measured using the Positive and Negative Syndrome Scale (PANSS) and were positively associated with exploration in their clinical samples. Moreover, Reddy & colleagues (2016) found that negative symptoms on the PANSS also were associated with greater exploration in those with psychosis. However, it is important to note that Cathomas & colleagues (2021) did not detect an association between depressive symptoms in their clinical sample, and Sheffield et al. (2022) did not detect an association between anxiety symptoms and exploration in their clinical samples.

Regarding symptom profiles across depression and anxiety, two studies (Aylward et al., 2019; Mukherjee et al., 2020) found a positive association between symptoms of depression and anxiety in their clinical population of individuals with major depressive disorder (MDD). Notably, both studies reported positive associations between exploration and depression, as well as exploration and anxiety in their MDD samples. This finding is consistent with studies finding that heightened anxiety was associated with greater exploration (Aberg et al., 2022; Smith et al., 2020). Moreover, Murphy & colleagues (2003) found that symptoms of anxiety were higher in their sample of individuals with MDD relative to healthy controls, although they did not formally test whether this translated to similar positive associations between anxiety symptoms and exploration. In contrast, one study (Brosolma et al., 2022) found no evidence for an association between exploration and depression (including symptoms of anhedonia) nor exploration and anxiety in their sample of individuals with MDD. Together, these studies could indicate the presence of common symptom profiles associated with biases in explore/exploit choices cross diagnostic boundaries, consistent with transdiagnostic approaches.

## Discussion

The present review examined whether explore/exploit decision-making is a transdiagnostic target across psychosis, depression, and anxiety. The findings suggest that there is currently insufficient evidence to conclude whether explore/exploit decision-making is a transdiagnostic target. Despite some evidence that psychosis, depression, and anxiety are associated with increased exploration, there was significant heterogeneity in methods used in this literature. Compared with the literature on depression and anxiety, studies examining explore/exploit biases in psychosis employed more consistent methods, and therefore, we are better able to draw conclusions about the association between psychosis and explore/exploit choices. We suggest that biases in explore/exploit choices can have profound, long-term consequences for individuals across the lifespan, as expressed by one of our lived experience advisors, “*There is probably a happy medium when it comes to explore/exploit decision-making, and mental illness can alter the ability to maintain this, which can affect a person long term.*” Therefore, this feature of decision-making should be further studied as a transdiagnostic target for mental health problems.

We originally predicted that psychosis and anxiety would be associated with heightened exploration, relative to those without these diagnoses, whereas we predicted that depression would be associated with reduced exploration compared to those without depression. There was moderate-strong evidence that psychosis was associated with increased exploration, consistent with our hypothesis. The evidence for anxiety partly supported our hypothesis, as half of the included studies examining anxiety found greater exploration in those with elevated levels of anxiety or a diagnosis of anxiety. Contrary to our prediction, several of the included studies found that individuals with depression explored more than those without depression. Our original hypothesis derived from evidence that depression is associated with reduced motivational drives (Grahek et al., 2019), which have been implicated in exploration choices (Bustamante et al., 2022). That we found depression was associated with heightened exploration could suggest that cognitive faculties other than motivation are responsible for explore/exploit decision-making are biased in mood disorders. One such cognitive faculty is individuals’ sensitivity to reward, which is diminished in individuals with depression (Halahakoon et al., 2020; Liu et al., 2016). Reduced reward sensitivity in those with depression could account for the heightened exploration observed in this population as participants are insensitive to the rewards available from exploiting a known option, instead selecting alternative options with unknown reward values (Costa et al., 2019). Computationally, reward sensitivity has been associated with the inverse temperature parameter (Browning et al., 2023), which was used to operationalise exploration in several of the studies included in the review. Indeed, reduced reward sensitivity also has been associated with psychosis (Saleh et al., 2023) and anxiety (LaFreniere & Newman, 2019; Potsch & Rief, 2023), indicating a mechanism that may underpin biases in explore/exploit choices across diagnostic boundaries.

The potential role of reward sensitivity in explore/exploit biases is notable, as there is evidence that these biases are modifiable through dopaminergic manipulation. Several studies that have demonstrated pharmacological agents are effective at modifying explore/exploit choices through increasing reward sensitivity (Chen et al., 2023; Cremer et al., 2022; Dubois et al., 2021; Sidorenko et al., 2023). For example, administering L-DOPA to healthy participants reduces their rates of exploration (Chakroun et al., 2020; Pessiglione et al., 2006). However, to date, no studies have attempted pharmacological manipulations to modify explore/exploit choices in the context of mental health problems, despite several of these pharmacological agents being relevant for the treatment of psychosis, depression, and anxiety.

The significant heterogeneity across this literature highlights the need for more precise specification regarding the feature(s) of exploration hypothesised to be impacted by psychopathology, such as whether exploration is driven by reduced reward sensitivity or an avoidance of uncertainty (Nussenbaum et al., 2023). In the current review, inconsistencies in the operationalisation of explore/exploit decision-making complicated our ability to draw conclusions about whether anxiety and depression were associated with biases in this behaviour. To robustly establish whether explore/exploit choices are biased in those with psychopathology, future work should refine measures of explore/exploit decision-making that can disentangle different cognitive faculties involved in these decisions and ensure these behavioural and computational measures are psychometrically valid and reliable over time (Pezzoli et al., 2023; Zorowitz & Niv, 2023) to allow systematic, longitudinal investigation of explore/exploit biases in psychopathology. Such methodological developments will be necessary to establish whether biases in explore/exploit decision-making are a cause or effect of mental health problems.

To address the heterogeneity with which mental health was recorded, future research should standardise which measures are used to assess symptomology and recruit large sample sizes with sufficient participants with high levels of symptomology. Studies that found a bias for heightened exploration in individuals with psychopathology were those that recruited participants with severe presentations (i.e., those with a diagnosed mental health problem), rather than community samples with lower levels of symptomatology. These findings could be considered consistent with transdiagnostic approaches, as there was heterogeneous presentation within diagnostic categories (i.e., between low-moderate and severe presentations) but some consistency across diagnostic categories insofar as those with clinical diagnoses typically explored more compared with healthy controls (Dalgleish et al., 2020; Krueger & Eaton, 2015). Moreover, there was some consistency in the symptom profiles that were associated with biases in explore/exploit decision-making, although these were not widely reported. To overcome these limitations, future research should explicitly examine this topic from a transdiagnostic perspective by recruiting samples that span across different diagnostic categories and symptom presentations.

Our lived experience advisors expressed the importance for future research to better establish links between explore/exploit tasks and real-world decisions. To our knowledge, no studies have examined the association between exploration preferences on explore/exploit paradigms and individuals’ proclivity to trial new experiences. Moreover, our lived experience advisors highlighted how explore/exploit choices may be affected by the individual’s cultural context where definitions of exploratory behaviour, and the extent to which exploration is promoted, may differ: “*further research should be done taking into account factors…such as cultural contexts.*” Indeed, all studies included in the review were conducted in high-income countries. Future research should consider how socioeconomic factors affect explore/exploit decision-making. To inform these important questions, our lived experience advisors suggested future research should be supplemented by a mixed-method approach to understand qualitative experiences of exploration and how they relate to mental health outcomes.

Examining how lab-based explore/exploit tasks relate to real world outcomes will be an important step in understanding how biases in explore/exploit choices are associated with the symptomology of psychosis, depression, and anxiety. Exploiting known options can lead people to reap rewards from their environment, whereas exploring novel opportunities can sometimes lead individuals to forfeit the most rewarding option. A bias to insufficiently exploit rewarding options could reflect symptoms commonly observed in psychosis, depression, and anxiety, such as the overestimation of the volatility of one’s surroundings in psychosis (Katthagen et al., 2022), reduced pursuit of pleasurable experiences in depression (Pizzagalli, 2014; Watson et al., 2020), or behavioural avoidance in individuals with anxiety (O’Brien et al., 2019; Zorowitz et al., 2020). Understanding the relationship between the symptomology of psychosis, depression, and anxiety and explore/exploit choices can contextualise commonly observed behaviours in individuals with these diagnoses.

Establishing whether explore/exploit biases are common across psychosis, depression, and anxiety has important implications for intervention development. We have suggested that explore/exploit choices may be biased in psychosis, depression, and anxiety due to altered reward sensitivity associated with these disorders. Several existing behavioural interventions focus on improving reward sensitivity as a method to reduce symptoms of psychopathology. For example, positive affect treatment is an intervention that encourages individuals to plan pleasurable activities and reinforce the positive mood affects associated with these experiences (Craske et al., 2016). Positive affect treatment has been demonstrated to improve symptoms of depression and anxiety at 6-month follow-up in clinical samples (Craske et al., 2019) and may be a promising intervention to improve explore/exploit biases in those with psychosis, depression, and anxiety. However, future research will need to explicitly examine whether the efficacy of positive affect treatment on psychopathology is mediated by an increased ability to exploit rewarding options. Such work should account for environmental exposures associated with biases in explore/exploit choices, such as experiences of childhood trauma (Lloyd et al., 2022). The potential for interventions to ameliorate biases in explore/exploit choices may be particularly impactful during adolescence, a period during which most mental health problems emerge (McGrath et al., 2023).

There are some important limitations to this review to consider. Although we examine explore/exploit decision-making as a transdiagnostic predictor of psychosis, depression, and anxiety, it is has been proposed that evidence of the “p-factor” is a statistical artefact rather than a genuine latent construct (Levin-Aspenson et al., 2021; Watts et al., 2019). We suggest that this debate can be partly informed by work to identify the existence (or lack thereof) of common, transdiagnostic cognitive biases that persist across diagnostic boundaries. Should common transdiagnostic cognitive biases be identified, this would lend some support to the notion of general vulnerability to psychopathology, and we suggest that explore/exploit decision-making should be empirically examined as one such bias. A methodological limitation of the reviewed studies is that it is not always possible to disentangle whether biases in explore/exploit decision-making in psychopathology are attributable to parameters measuring exploration alone or an interaction between parameters. For example, Martinelli et al. (2018) found that individuals with a diagnosis of Schizophrenia also exhibited differences in the learning rate parameter, which is responsible for updating the expected value of available options. Biases in the learning rate parameter meant that individuals failed exploit high-reward options they had already samples. Therefore, it is not always possible to conclude whether exploration arises from a bias to sample available options or an inability to identify and exploit high-reward options.

In summary, the current review examined explore/exploit decision-making as a transdiagnostic target for psychosis, depression, and anxiety. Findings were mixed regarding the association between explore/exploit choices and depression and anxiety, although we have suggested that such inconsistencies could be attributed to methodological differences in the literature. In contrast, the findings related to psychosis supported the view that those with psychosis explore more than those without psychosis. We have suggested that there is currently insufficient evidence to conclude whether explore/exploit decision-making is a transdiagnostic target for psychosis, depression, and anxiety, but we encourage future research in this area. Biases in explore/exploit decision-making may explain symptoms associated with psychosis, depression, and anxiety, and we have proposed that existing interventions might be promising routes to ameliorate biases in explore/exploit decision-making associated with psychopathology. These views were endorsed by our lived experience advisory groups, who highlighted the need to support those with psychosis, depression, and anxiety to identify and exploit rewarding opportunities available to them. Altogether, explore/exploit decision-making is a feature of cognition that has the potential to significantly improve the lives of individuals if targeted for intervention.

## Supplementary Information

 Below is the link to the electronic supplementary material.

Supplementary file1 (DOCX 6.78 MB)

## Declarations

### Open practices statement

This systematic review was pre-registered at: https://osf.io/tmcxz.
