# Supplementary material for: Reviewing explore/exploit decision-making as a transdiagnostic target for psychosis, depression, and anxiety
Source: Cogn Affect Behav Neurosci. 2024 Apr 23;24(5):793–815. doi: 10.3758/s13415-024-01186-9 (PMC11390819; doi:10.3758/s13415-024-01186-9)
Supplement: Supplementary file 1 — Supplementary file1 (DOCX 6.78 MB) [file 13415_2024_1186_MOESM1_ESM.docx]

**Supplementary Materials: Co-Production Output**

**Miro board examining the links between exploration, exploitation, and mental health.**

**
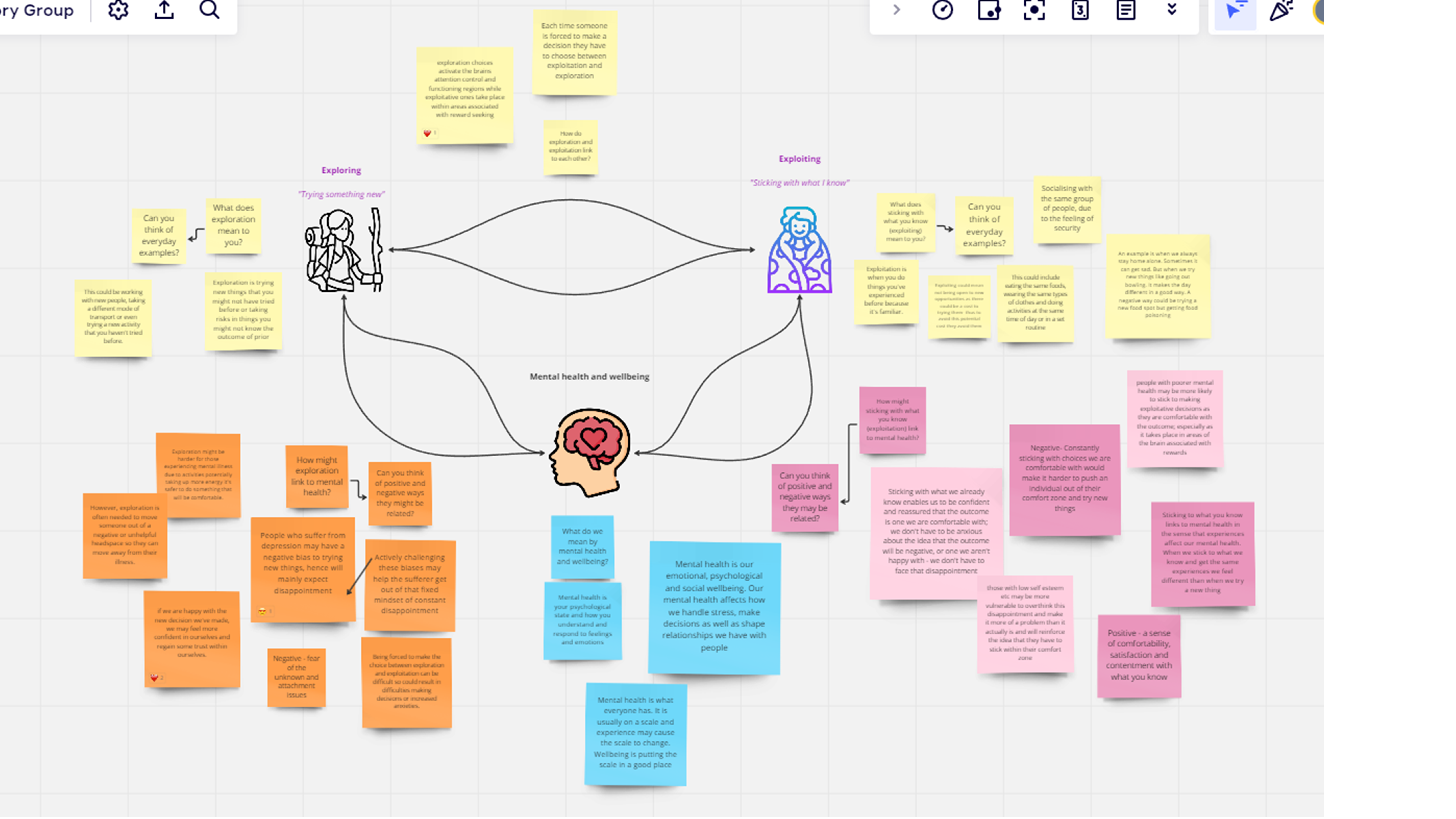
**
